# Association of body mass index with perioperative blood transfusion and short-term clinical outcomes in patients undergoing isolated coronary artery bypass grafting

**DOI:** 10.1186/s12871-023-02329-0

**Published:** 2023-11-03

**Authors:** Jie Gao, Hongwen Ji

**Affiliations:** 1 Department of Anesthesiology, Fuwai Hospital, Chinese Academy of Medical Science and Peking Union Medical College, Beijing, China; 2 Department of Transfusion Medicine, Fuwai Hospital, Chinese Academy of Medical Science and Peking Union Medical College, Beijing, China

**Keywords:** Body mass index, Blood transfusion, Clinical outcome, Coronary artery bypass grafting, Cardiopulmonary bypass

## Abstract

**Background:**

Few studies have considered outcomes among low body mass index (BMI) cohorts undergoing coronary artery bypass grafting (CABG). This study aims to investigate the effects of low body weight on blood transfusion and perioperative outcomes in patients undergoing isolated CABG.

**Methods:**

This retrospective study enrolled consecutive cases from a single-center between January 2008 and December 2018. Low body weight/underweight was defined as a BMI < 18.5 kg/m², while normal BMI was defined as 18.5 ≤ BMI < 24.0 kg/m². The primary endpoint was the perioperative red blood cell (RBC) transfusion rate. Secondary endpoints include platelet and plasma transfusion rates, transfusion volume for all blood components, hospital length of stay, and the occurrence of adverse events including prolonged mechanical ventilation, re-intubation, re-operation, acute kidney injury, and 30-day all-cause mortality.

**Results:**

A total of 7,620 patients were included in this study. After 1:1 propensity score matching, 130 pairs were formed, with 61 pairs in the on-pump group and 69 pairs in the off-pump group. Baseline characteristics were comparable between the matched groups. Low body weight independently increased the risk of RBC transfusion (on-pump: OR = 3.837, 95% CI = 1.213–12.144, p = 0.022; off-pump: OR = 3.630, 95% CI = 1.875–5.313, p < 0.001). Moreover, within the on-pump group of the original cohort, BMI of < 18.5 kg/m² was independently correlated with increased risk of re-intubation (OR = 5.365, 95% CI = 1.159 to 24.833, p = 0.032), re-operation (OR = 4.650, 95% CI = 1.019 to 21.210, p = 0.047), and 30-day all-cause mortality (OR = 10.325, 95% CI = 2.011 to 53.020, p = 0.005).

**Conclusion:**

BMI < 18.5 kg/m² was identified as an independent risk factor for increased perioperative RBC transfusion rate in patient underwent isolated CABG with or without CPB. Only on-pump underweight patients in the original cohort exhibited an increased risk for re-intubation, re-operation, and 30-day all-cause mortality. Physicians and healthcare systems should consider these findings to improve management for this population.

## Introduction

Obesity, measured by body mass index (BMI), is a significant health issue associated with an increased burden of cardiovascular disease (CVD) risk factors and reduced life expectancy [1]. The relationship between obesity and CVD is well-established, including conditions such as acute coronary syndrome, acute myocardial infarction, heart failure, and coronary artery disease [2–5]. This association is attributed to various underlying mechanisms, including chronic tissue hypoxia, hyperinsulinemia, and inflammatory responses triggered by tissue lipid peroxidation [6].

However, intriguingly, studies have reported an unexpected phenomenon known as the “obesity paradox“ [1, 7]. Despite obesity being a prominent risk factor for the development and prognosis of CVD, it is paradoxically linked to reduced mortality rates and fewer respiratory complications following cardiac surgery [8]. Additionally, obese individuals tend to require fewer allogeneic blood transfusions [9]. In recent years, the focus of research has predominantly centered on the clinical outcomes of obese patients, while the specific clinical outcomes of underweight individuals have received considerably less attention. In contrast to obesity, low body weight among adults is often insufficiently addressed in clinical practice. Popular education programs promoting coronary heart disease prevention and treatment tend to prioritize weight control, leading individuals to erroneously believe that a low BMI equates to lower blood lipid levels and a decreased risk of atherosclerosis [10]. Consequently, the potential risks associated with low body weight are frequently overlooked.

Numerous studies have demonstrated an inverse J-shaped relationship between BMI and cardiovascular mortality in patients with coronary artery disease, indicating that both underweight and overweight/obese patients may exhibit higher rates of cardiovascular mortality [11, 12]. However, the majority of research have primarily focused on mortality as a severe adverse event following coronary artery bypass surgery (CABG), which has become less prevalent due to advancements in surgical techniques [13]. Moreover, despite the significance of mortality as a clinical outcome, there remains a dearth of comprehensive understanding concerning perioperative adverse events in patients undergoing CABG.

Allogeneic blood transfusions have known detrimental effects on the recovery and short- and long-term prognosis of both cardiac and non-cardiac patients [14, 15]. Studies have noted a higher need for blood transfusion in underweight populations, particularly during open heart surgery, possibly due to lower preoperative hemoglobin levels and increased blood dilution associated with cardiopulmonary bypass (CPB) [9, 13]. However, the mechanisms underlying these associations and their potential causes remain incompletely understood. Existing analyses in this field have primarily concentrated on the impact of on-pump CABG surgery, which can induce blood dilution and pose a substantial challenge to individuals with lower blood volume, such as those with low body weight. The specific influence of low body weight on off-pump CABG surgery has not been extensively investigated, and it is uncertain whether the effects are comparable to those observed in on-pump procedures.

Studies have indicated that the prevalence of BMI < 18.5 kg/m^2^ is generally higher among Asian populations compared to most Western populations [16, 17]. This disparity in prevalence underscores the necessity of examining the specific impact of low BMI in patients undergoing CABG. In China, particularly, the large population base contributes to a relatively substantial number of underweight individuals, despite the low incidence of low BMI within the coronary heart disease population. However, due to the limited number of patients with low BMI in the coronary heart disease cohort, they are often combined with patients possessing normal BMI. This grouping approach obscures the true impact of BMI differences on outcomes, as each group comprises individuals with highly diverse BMI ranges.

Given the current dearth of research regarding the association between blood transfusion and other perioperative outcomes in underweight patients undergoing CABG, as well as the intricate relationship between BMI and surgical outcomes in different CABG procedures, the objective of this study is to investigate the impact of BMI on blood transfusion as well as perioperative outcomes in patients undergoing isolated CABG.

## Materials and methods

### Study population and data collection

**Fig. 1:**
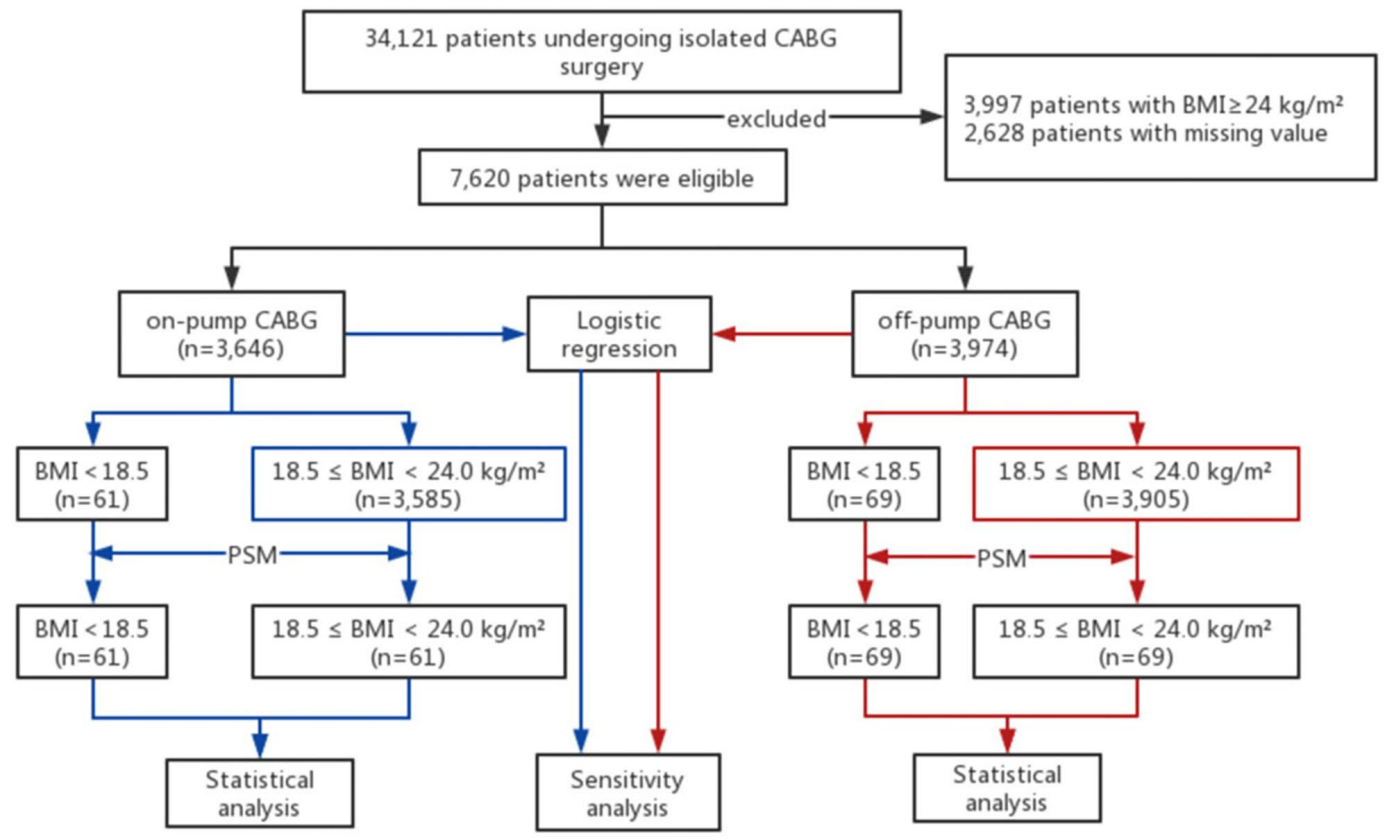
The flow chart of different BMI categories in patients undergoing isolated coronary artery bypass grafting surgery. The flow chart outlines the steps of the study, including the selection of patients, BMI categorization, and the application of propensity score matching PSM to create matched cohorts. CABG, coronary artery bypass grafting; BMI, body mass index; PSM, propensity score matching

This study was conducted in accordance with the ethical principles outlined in the Declaration of Helsinki and received approval from the institutional review board of Fuwai Hospital, Beijing, China. The study population comprised a total of 34,121 consecutive patients who underwent isolated CABG at Fuwai Hospital between 1 and 2008 and 31 December 2018. To ensure the homogeneity of the study population and enhance the robustness of data analysis, patients who underwent concomitant cardiovascular surgical procedures were excluded. Additionally, participants with BMI ≥ 24 kg/m^2^ (n = 3997) and those with missing values (n = 2628) were further excluded from the study. As a result, a total of 7,620 participants remained for data analysis. A detailed flowchart illustrating the participant selection process can be found in Fig. 1.

Patient data were collected from the electronic medical records of the hospital, encompassing various aspects such as baseline characteristics, diagnoses, operation details, perioperative laboratory test results, and postoperative data, especially perioperative transfusion utilization and clinical outcomes. Preexisting medical conditions were identified using the International Classification of Diseases, 9th Revision (ICD-9) codes. The names of the surgical procedures were extracted from discharge diagnoses and verified through both surgical and anesthesia records. To ensure privacy and maintain data anonymization, the original researchers encoded participant identities into untraceable codes.

### Endpoints and definitions

The primary endpoint of the study is the allogeneic red blood cell (RBC) transfusion rate during the perioperative period. Secondary endpoints include the rates of platelet and plasma transfusion, the volume of allogeneic transfusion for all blood components, hospital length of stay, and the occurrence of adverse events such as prolonged mechanical ventilation, re-intubation, re-operation, acute kidney injury (AKI), and 30-day all-cause mortality.

BMI was calculated as the weight in kilograms divided by the square of the height in meters (kg/m^2^). Among them, underweight is defined as BMI < 18.5 kg/m^2^, while normal BMI is defined as 18.5 ≤ BMI < 24.0 kg/m², according to the World Health Organization (WHO) classification and Work Group on Obesity in China (WGOC) classification [18]. Prolonged mechanical ventilation is defined as mechanical ventilation exceeding a duration of 48 h. AKI is diagnosed following the criteria of the Acute Kidney Injury Network [19]. The calculation of the change in systolic blood pressure (SBP) following the administration of general anesthesia was calculated as: (Difference in SBP before and after anesthesia induction / pre-induction SBP) * 100%.

### Statistical analysis

Normal distribution of continuous variables was tested using the Shapiro-Wilks test. To ensure consistency, continuous variables were presented as mean ± standard deviation if the variables follow a normal distribution, otherwise median and interquartile range (IQR), categorical variables were presented as numbers and percentages. Continuous variables with normal distribution were compared by Student’s t-test between two groups. Continuous variables with skewed distribution were compared by Mann-Whitney U test. We compared categorical variables with the χ^2^ test or Fisher’s exact test. Since we have a large enough sample size, patients with missing data were excluded.

Propensity score matching was employed to address the baseline differences between two groups. BMI was served as the grouping variable, and covariates exhibiting differences between the groups were determined by clinical expertise were considered (Table 1). Propensity scores were computed based on the selected variables and utilized to create a matched group of subjects with comparable baseline characteristics. The 1:1 exact matching method was preferred, and the nearest neighbor matching method without replacement was employed, using a caliper value of 0.02. A total of 130 pairs of patients were successfully matched, comprising 61 pairs in the on-pump group and 69 pairs in the off-pump group. The balance between groups before and after matching was evaluated using p-values, whereby a p-value > 0.05 indicated no statistically significant differences in the distribution of covariates between the two groups.

**Table 1:** Variables in the propensity score matching

Order	Variables
1	Age
2	Gender
3	LVEF < 40%
4	Hemoglobin (g/L)
5	Diabetes mellitus
6	Carotid stenosis
7	Hypertension
8	Hyperlipidemia
9	Chronic obstructive pulmonary disease
10	Peripheral artery disease
11	Chronic kidney disease
12	Atrial fibrillation
13	Pulmonary-arterial hypertension
14	Previous cardiac surgery
15	Previous CVA
16	Previous PCI
17	Duration of CPB (min)
18	Duration of operation (min)
19	Emergency surgery
20	Numbers of bridging vessels

Abbreviations: LVEF, left ventricular ejection fractions; CVA, Cerebrovascular accident; PCI, percutaneous coronary intervention; CPB, cardiopulmonary bypass

Subsequently, statistical analysis was performed on the matched variables to estimate the effects of BMI on transfusion and clinical outcomes. To evaluate the robustness of the results, a sensitivity analysis was conducted by including the original cohort. Variables that showed multicollinearity, as determined by a variance inflation factor greater than 10, were subsequently eliminated. Furthermore, based on clinical expertise, relevant variables identified in the univariate regression analysis were included in a multivariate regression analysis to construct a binary logistic regression model.

Additionally, a post-hoc analysis was conducted to explore potential factors underlying the observed findings. This analysis involved examining the clinical and laboratory indicators of the 130 successfully matched pairs of patients. All statistical analyses were performed using SPSS 26.0 software (SPSS Inc., Chicago, IL, USA). A bilateral p-value of 0.05 was considered statistically significant.

## Results

### Characteristics of the study population

A total of 31,493 patients met the inclusion and exclusion criteria, and 130 pairs were created after the propensity score matching, including 61 pairs in the on-pump group and 69 pairs in the off-pump group. In the on-pump group, patients with a BMI < 18.5 kg/m² exhibited order age, higher prevalence of chronic obstructive pulmonary disease (COPD), congestive heart failure (CHF), and pulmonary arterial hypertension (PAH), and lower prevalence of diabetes, hypertension, and hyperlipidemia, along with significant differences in the number of bridging vessels between the two groups (all p < 0.05, Table 2). After matching, no statistical differences were observed for all variables (all p > 0.05, Table 2). Similarly, in the off-pump group, patients with a BMI < 18.5 kg/m² were characterized by older age, fewer males, and a lower prevalence of diabetes before matching (all p < 0.05, Table 3). After matching, there were no statistical differences observed for all variables (all p > 0.05, Table 3).

**Table 2:** Demographic and clinical variables before and after propensity score matching in on-pump CABG group

Variables	Before matching			After matching		
	BMI<18.5 kg/m^2^ (n = 61)	18.5 ≤ BMI < 24 kg/m^2^ (n = 3585)	p value	BMI<18.5 kg/m^2^ (n = 61)	18.5 ≤ BMI < 24 kg/m^2^ (n = 61)	p value
Age (y), mean ± SD	64.92 ± 7.22	62.37 ± 8.11	**0.015**	64.92 ± 7.22	62.80 ± 8.65	0.145
Gender male, n (%)	39 (63.9)	2626 (73.2)	0.104	39 (63.9)	45 (73.8)	0.241
Smoking history, n (%)	22 (52.5)	1760 (49.1)	0.602	32 (52.5)	38 (62.3)	0.272
Drinking history, n (%)	15 (24.6)	611 (17.0)	0.121	13 (21.3)	15 (24.6)	0.667
LV dysfunction (ejection fraction < 40%), n (%)	3 (4.9)	119 (3.3)	0.491	3 (4.9)	1 (1.6)	0.619
HGB (g/L), mean ± SD	132.58 ± 16.50	134.23 ± 13.73	0.440	132.58 ± 16.50	133.93 ± 15.22	0.639
**Preexisting medical conditions, n (%)**						
Diabetes mellitus	11 (18)	1111 (31.0)	**0.030**	11 (18.0)	15 (24.6)	0.377
Carotid stenosis	0 (0)	72 (2.0)	0.264	0 (0)	3 (4.9)	0.244
Hypertension	23 (37.7)	1856 (51.8)	**0.029**	23 (37.7)	32 (52.5)	0.102
Hyperlipidemia	24 (39.3)	1946 (54.3)	**0.020**	24 (39.3)	34 (55.7)	0.070
COPD	5 (8.2)	118 (3.3)	**0.035**	5 (8.2)	5 (8.2)	1.000
Peripheral artery disease	2 (3.3)	74 (2.1)	0.510	2 (3.3)	0 (0)	0.154
Chronic kidney disease	0 (0)	32 (0.9)	0.459	0 (0)	1 (1.6)	1.000
Atrial fibrillation	1 (1.6)	47 (1.3)	0.832	1 (1.6)	1 (1.6)	1.000
Chronic heart failure	1 (1.6)	5 (0.1)	**0.004**	1 (1.6)	0 (0)	1.000
Pulmonary-arterial hypertension	2 (3.3)	14 (0.4)	**0.001**	2 (3.3)	2 (3.3)	1.000
Previous CVA	10 (16.4)	418 (11.7)	0.255	10 (16.4)	10 (16.4)	1.000
Previous myocardial infarction	19 (31.1)	945 (26.4)	0.400	19 (31.1)	14 (23.0)	0.308
Previous PCI	3 (4.9)	353 (9.8)	0.198	3 (4.9)	8 (13.1)	0.114
Previous cardiac surgery	1 (1.6)	80 (2.2)	0.756	1 (1.6)	3 (4.9)	0.619
**Operation characteristics**						
Duration of CPB (min), mean ± SD	138.00 ± 25.23	137.46 ± 28.59	0.884	138.00 ± 25.23	140.56 ± 30.33	0.613
Duration of operation (min), mean ± SD	318.63 ± 81.00	311.22 ± 79.83	0.473	318.63 ± 81.00	311.47 ± 74.98	0.614
Emergency surgery, n (%)	2 (3.3)	101 (2.8)	0.829	2 (3.3)	0 (0)	0.496
Numbers of bridging vessels, n (%)						
one	4 (6.5)	49 (1.4)	**0.001**	4 (6.5)	0 (0)	0.119
two	7 (11.4)	400 (11.2)	0.938	7 (11.5)	7 (11.5)	1.000
three	25 (40.9)	1596 (44.5)	0.582	25 (41.0)	24 (39.3)	0.853
three more	25 (40.9)	1540 (43.0)	0.758	25 (41.0)	30 (49.2)	0.363

Abbreviations: BMI, body mass index; SD, standard deviation; LV, left ventricular; HGB, hemoglobin; COPD, chronic obstructive pulmonary disease; CVA, Cerebrovascular accident; PCI, percutaneous coronary intervention; CPB, cardiopulmonary bypass

**Table 3:** Demographic and clinical variables before and after propensity score matching in off-pump CABG group

Variables	Before matching			After matching		
	BMI<18.5 kg/m^2^ (n = 69)	18.5 ≤ BMI < 24 kg/m^2^ (n = 3905)	p value	BMI<18.5 kg/m^2^ (n = 69)	18.5 ≤ BMI < 24 kg/m^2^ (n = 69)	p value
Age (y), mean ± SD	65.87 ± 7.95	63.04 ± 8.33	0.005	65.87 ± 7.95	65.26 ± 9.06	0.676
Gender male, n (%)	35 (50.7)	2845 (72.9)	<0.001	35 (50.7)	40 (58.0)	0.393
Smoking history, n (%)	31 (44.9)	2037 (52.2)	0.233	31 (44.9)	41 (59.4)	0.088
Drinking history, n (%)	8 (11.6)	746 (19.1)	0.115	8 (11.6)	14 (20.3)	0.163
LV dysfunction (ejection fraction < 40%), n (%)	3 (4.3)	91 (2.3)	0.274	3 (4.3)	1 (1.4)	0.310
HGB (g/L), mean ± SD	135.63 ± 15.49	135.64 ± 13.93	0.996	135.63 ± 15.49	135.82 ± 15.22	0.945
**Preexisting medical conditions, n (%)**						
Diabetes mellitus	10 (14.5)	1201 (30.8)	0.004	10 (14.5)	19 (27.5)	0.060
Carotid stenosis	3 (4.3)	102 (2.6)	0.373	3 (4.3)	5 (7.2)	0.466
Hypertension	33 (47.8)	2022 (51.8)	0.515	33 (47.8)	35 (50.7)	0.733
Hyperlipidemia	31 (44.9)	2214 (56.7)	0.051	31 (44.9)	40 (58.0)	0.125
COPD	4 (5.8)	125 (3.2)	0.228	4 (5.8)	6 (8.7)	0.511
Peripheral artery disease	1 (1.4)	96 (2.5)	0.590	1 (1.4)	2 (2.9)	0.559
Chronic kidney disease	1 (1.4)	37 (0.9)	0.671	1 (1.4)	0 (0)	1.000
Atrial fibrillation	2 (2.9)	65 (1.7)	0.430	2 (2.9)	3 (4.3)	1.000
Chronic heart failure	0 (0)	5 (0.1)	1.000	0 (0)	0 (0)	/
Pulmonary-arterial hypertension	0 (0)	8 (0.2)	0.707	0 (0)	0 (0)	/
Previous CVA	9 (13.0)	432 (11.1)	0.604	9 (13.0)	11 (15.9)	0.629
Previous myocardial infarction	18 (26.1)	938 (24.0)	0.691	18 (26.1)	11 (15.9)	0.144
Previous PCI	8 (11.6)	394 (10.1)	0.681	8 (11.6)	12 (17.4)	0.333
Previous cardiac surgery	0 (0)	66 (1.7)	0.276	0 (0)	0 (0)	/
**Operation characteristics**						
Duration of operation (min), mean ± SD	293.10 ± 94.50	292.79 ± 86.26	0.977	293.10 ± 94.50	295.39 ± 88.68	0.884
Emergency surgery, n (%)	3 (4.3)	121 (3.1)	0.554	3 (4.3)	2 (2.9)	0.649
Numbers of bridging vessels, n (%)						
one	5 (7.2)	385 (9.9)	0.470	5 (7.2)	7 (10.1)	0.546
two	16 (23.1)	587 (15.0)	0.061	16 (23.1)	11 (15.9)	0.283
three	28 (40.5)	1633 (41.8)	0.836	28 (40.5)	27 (39.1)	0.862
three more	20 (28.9)	1300 (33.3)	0.452	20 (28.9)	24 (34.7)	0.465

Abbreviations: BMI, body mass index; SD, standard deviation; LV, left ventricular; HGB, hemoglobin; COPD, chronic obstructive pulmonary disease; CVA, Cerebrovascular accident; PCI, percutaneous coronary intervention; CPB, cardiopulmonary bypass

### Transfusion utilization

#### For patients underwent on-pump CABG

After propensity score matching, significant differences were observed between the two groups in the RBC, plasma, platelet, and total transfusion rate (RBC: 37.7% vs. 21.3%, p = 0.047; FFP: 31.1% vs. 9.8%, p = 0.004; PLT: 42.6% vs. 24.6%, p = 0.035). However, there were no significant differences in the transfusion volumes of each blood component (RBC: 2.34 ± 4.62 vs. 1.25 ± 3.80; FFP: 193.44 ± 332.60 vs. 111.48 ± 445.01; PLT: 0.26 ± 1.26 vs. 0.30 ± 0.26; all p > 0.05; Table 4). Patients with low BMI, compared to normal-BMI patients, exhibited a significantly increased requirement for transfusion of RBCs, plasma, platelets, and overall transfusions (RBC: OR = 3.837, 95% CI = 1.213–12.144, p = 0.022; FFP: OR = 10.135, 95% CI = 1.693–60.627, p = 0.011; PLT: OR = 3.208, 95% CI = 1.104–9.137, p = 0.032; any: OR = 3.208, 95% CI = 1.104–9.137, p = 0.032; Table 4). These associations were also observed in the original cohort.

**Table 4:** Comparison of transfusion utilization between different BMI groups in on-pump CABG surgery

Variables	PSM				Logistic			
	BMI<18.5 kg/m^2^ (n = 61)	18.5 ≤ BMI < 24 kg/m^2^ (n = 61)	MD or OR (95%CI)	p value	BMI<18.5 kg/m2 (n = 61)	18.5 ≤ BMI < 24 kg/m^2^ (n = 3585)	OR (95%CI)	p value
**Transfusion requirement (quantity if used and percentage of usage)**								
Red blood cell (u), mean ± SD	2.34 ± 4.62	1.25 ± 3.80	1.180 (-0.419 ~ 2.615)	0.154	2.34 ± 4.62	1.07 ± 2.61	/	/
n (%)	23 (37.7)	13 (21.3)	3.837 (1.213 ~ 12.144)	**0.022**	23 (37.7)	695 (19.4)	2.019 (1.232 ~ 3.609)	**0.007**
Plasma (mL), mean ± SD	193.44 ± 332.60	111.48 ± 445.01	81.970 (-58.990 ~ 22.924)	0.252	193.44 ± 332.60	105.10 ± 332.60	/	/
n (%)	19 (31.1)	6 (9.8)	10.135 (1.693 ~ 60.627)	**0.011**	19 (31.1)	480 (13.4)	2.973 (1.700 ~ 5.200)	**<0.001**
Platelets (u), mean ± SD	0.26 ± 1.26	0.30 ± 0.26	0.230 (-0.100 ~ 0.566)	0.169	0.26 ± 1.26	0.07 ± 0.78	/	**/**
n (%)	26 (42.6)	15 (24.6)	3.208 (1.104 ~ 9.137)	**0.032**	26 (42.6)	914 (25.5)	1.913 (1.140 ~ 3.208)	**0.014**
Any, n (%)	26 (42.6)	15 (24.6)	3.208 (1.104 ~ 9.137)	**0.032**	26 (42.6)	914 (25.5)	1.913 (1.140 ~ 3.208)	**0.014**

Abbreviations: PSM, propensity score matching; BMI, body mass index; MD, mean difference; OR, odds ratio; CI, confidence interval; SD, standard deviation

#### For patients underwent off-pump CABG

After conducting propensity score matching, a statistically significant difference was observed between the two groups in the RBC and total transfusion rate (RBC: 39.1% vs. 15.9%, p = 0.002; any: 42.0% vs. 23.2%, p = 0.018). However, there was no significant difference between the groups in the plasma and platelet transfusion rate (FFP: 14.5% vs. 10.1%, p = 0.437; PLT: 4.3% vs. 0%, p = 0.245). Only the RBC transfusion volume showed a statistically significant difference between the groups (1.26 ± 1.97 vs. 0.44 ± 1.08, p = 0.003; Table 5), whereas no similar association was observed for plasma and platelet transfusions. After matching, underweight patients exhibited a significantly increased requirement of transfusion for RBCs compared to the matched control group (OR = 3.630, 95% CI = 1.326–9.937, p = 0.012; Table 5). In the original cohort, underweight patients exhibited a significantly increased requirement for transfusion of RBCs (OR = 3.630, 95% CI = 1.875–5.313, p < 0.001; Table 5), plasma (OR = 1.334, 95% CI = 0.674–2.641, p = 0.048; Table 5), platelets (OR = 4.028, 95% CI = 1.135–14.303, p = 0.031; Table 5), and the total transfusion (OR = 2.261, 95% CI = 1.383–3.696, p = 0.001; Table 5).

**Table 5:** Comparison of transfusion utilization between different BMI groups in off-pump CABG surgery

Variables	PSM				Logistic			
	BMI<18.5 kg/m^2^ (n = 69)	18.5 ≤ BMI < 24 kg/m^2^ (n = 69)	MD or OR (95%CI)	p value	BMI<18.5 kg/m2 (n = 69)	18.5 ≤ BMI < 24 kg/m^2^ (n = 3905 )	OR (95%CI)	p value
**Transfusion requirement (quantity if used and percentage of usage)**								
Red blood cell (u), mean ± SD	1.26 ± 1.97	0.44 ± 1.08	0.826 (0.291 ~ 1.362)	**0.003**	1.26 ± 1.97	0.71 ± 2.07	/	/
n (%)	27 (39.1)	11 (15.9)	3.630 (1.326 ~ 9.937)	**0.012**	27 (39.1)	504 (12.9)	3.156 (1.875 ~ 5.313)	**<0.001**
Plasma (mL), mean ± SD	86.96 ± 287.95	46.38 ± 146.11	40.580 (-36.534 ~ 117.693)	0.299	86.96 ± 287.95	82.38 ± 237.164	/	/
n (%)	10 (14.5)	7 (10.1)	1.201 (0.036 ~ 4.228)	0.778	10 (14.5)	492 (12.6)	1.334 (0.674 ~ 2.641)	**0.048**
Platelets (u), mean ± SD	0.07 ± 0.40	0 ± 0	0.072 (-0.023~-0.167)	0.133	0.07 ± 0.40	0.02 ± 0.25	/	/
n (%)	3 (4.3)	0 (0)	/	/	3 (4.3)	40 (1.0)	4.028 (1.135 ~ 14.303)	**0.031**
Any, n (%)	29 (42.0)	16 (23.2)	2.462 (0.955 ~ 6.349)	0.062	29 (42.0)	812 (20.8)	2.261 (1.383 ~ 3.696)	**0.001**

Abbreviations: PSM, propensity score matching; BMI, body mass index; MD, mean difference; OR, odds ratio; CI, confidence interval; SD, standard deviation

### Clinical outcomes

In the on-pump group, after propensity score matching, there were no significant differences in the incidence of prolonged mechanical ventilation, re-intubation, re-operation, AKI, 30-day all-cause mortality, and hospital length of stay between the two groups (all p > 0.05). No deaths were observed in the matched control group. In the matched cohort, there was no evidence of an increased risk of perioperative adverse events associated with a BMI < 18.5 kg/m² (p > 0.05). However, in the original cohort, BMI < 18.5 kg/m² was identified as an independent risk factor for re-intubation, re-operation and 30-day all-cause mortality (OR = 5.365, 95% CI = 1.159 to 24.833, p = 0.032; OR = 4.650, 95% CI = 1.019 to 21.210, p = 0.047; OR = 10.325, 95% CI = 2.011 to 53.020, p = 0.005; Table 6).

**Table 6:** Comparison of clinical outcomes among different BMI groups in patients undergoing isolated CABG with/without CPB. Abbreviations: PSM, propensity score matching; BMI, body mass index; MD, mean difference; OR, odds ratio; CI, confidence interval; SD, standard deviation

Variables	PSM				Logistic			
	BMI<18.5 kg/m^2^	18.5 ≤ BMI < 24 kg/m^2^	MD or OR (95%CI)	p value	BMI<18.5 kg/m^2^	18.5 ≤ BMI < 24 kg/m^2^	OR (95%CI)	p value
**On-pump group (n = 61)**								
**On-pump group**	n = 61	n = 61			n = 61	n = 3585		
Postoperative complications, n (%)								
Prolonged mechanical ventilation	3 (4.9)	3 (4.9)	2.073 (0.149 ~ 28.839)	0.587	3 (4.9)	75 (2.1)	1.065 (0.247 ~ 4.595)	0.932
Re-intubation	3 (4.9)	3 (4.9)	2.907 (0.239 ~ 35.321)	0.402	3 (4.9)	21 (0.6)	5.365 (1.159 ~ 24.833)	**0.032**
Re-operation due to major hemorrhage or cardiac tamponade	2 (3.3)	3 (4.9)	3.007 (0.249 ~ 36.261)	0.386	2 (3.3)	34 (0.9)	4.650 (1.019 ~ 21.210)	**0.047**
Acute kidney injury	2 (3.3)	4 (6.6)	0.118 (0.001 ~ 48.384)	0.486	2 (3.3)	45 (1.3)	0.727 (0.121 ~ 4.366)	0.728
30-day all-cause mortality	2 (3.3)	0 (0)	/	/	2 (1.6)	5 (0.1)	10.325 (2.011 ~ 53.020)	**0.005**
Hospital length of stay (d), mean ± SD	16.57 ± 8.20	15.75 ± 4.17	0.820 (-1.521 ~ 3.161)	0.488	16.57 ± 8.20	15.77 ± 5.47	/	/
**Off-pump group**	n = 69	n = 69			n = 69	n = 3905		
Postoperative complications, n (%)								
Re-operation due to major hemorrhage or cardiac tamponade	1 (1.4)	0 (0)	/	/	1 (1.4)	11 (0.3)	6.636 (0.809 ~ 54.435)	0.078
Hospital length of stay (d), mean ± SD	15.55 ± 6.10	13.83 ± 4.63	1.725 (-0.099 ~ 3.549)	0.064	15.55 ± 6.10	15.41 ± 5.33	/	/

Abbreviations: PSM, propensity score matching; BMI, body mass index; MD, mean difference; OR, odds ratio; CI, confidence interval; SD, standard deviation

In the off-pump group, both the matched control and matched underweight groups exhibited no instances of prolonged mechanical ventilation, re-intubation, re-operation, or AKI. Only 1 case of AKI (1.4%) was recorded in the matched control group, along with 1 instance of re-operation (1.4%) in the matched underweight group. Hospital length of stay showed no significant difference between the two groups. In the original cohort, only 30-day all-cause mortality was assessed, as no other complications were observed in the underweight patient population, and a BMI < 18.5 kg/m² was not identified as an independent risk factor for 30-day all-cause mortality (OR = 6.636, 95% CI = 0.809 to 54.435, p = 0.078; Table 6).

### Post-hoc analysis

The post-hoc analysis of this study included a total of 130 pairs of successfully matched patients. Comparisons with the matched control group revealed significantly lower levels of prealbumin (226.322 ± 46.06 vs. 237.76 ± 46.03, p = 0.046), albumin (41.47 ± 4.74 vs. 43.94 ± 4.08, p<0.001), albumin/globulin (A/G) ratio (1.64 ± 0.44 vs. 1.82 ± 0.39, p = 0.001), and post-induction SBP (96.42 ± 15.04 vs. 112.02 ± 17.20, p<0.001) in the underweight group. Additionally, the SBP change rate was greater in the underweight group (34.31 ± 8.47 vs. 23.06 ± 8.14, p<0.001). However, no significant differences were observed between two groups regarding pre-induction SBP, total protein, and total cholesterol (all p>0.05; Table 7).

**Table 7:** Post-hoc analysis of laboratory and clinical indicators during operation in different BMI groups in patients undergoing isolated CABG.

Variables	BMI<18.5 kg/m^2^ (n = 130)	18.5 ≤ BMI < 24 kg/m^2^ (n = 130)	MD (95%CI)	p value
Prealbumin (mg/L), mean ± SD	226.322 ± 46.06	237.76 ± 46.03	−11.438 (−22.684 ~−0.192)	**0.046**
Total protein (g/L), mean ± SD	68.44 ± 8.85	69.13 ± 6.77	−0.690 (−2.614 ~ 1.234)	0.447
Albumin (g/L), mean ± SD	41.47 ± 4.74	43.94 ± 4.08	−2.470 (−3.550~−1.390)	**<0.001**
Total cholesterol (mmol/L), mean ± SD	3.80 ± 1.20	3.90 ± 1.39	−0.100 (−0.417 ~ 0.217)	0.520
Albumin/globulin ratio, mean ± SD	1.64 ± 0.44	1.82 ± 0.39	−0.180 (−0.282~−0.078)	**0.001**
Pre-induction SBP (mmHg), mean ± SD	148.20 ± 23.64	146.35 ± 22.04	1.850 (−3.732 ~ 7.432)	0.515
Post-induction SBP (mmHg), mean ± SD	96.42 ± 15.04	112.02 ± 17.20	−15.600(−19.546~−11.654)	**<0.001**
SBP change rate (%), mean ± SD	34.31 ± 8.47	23.06 ± 8.14	11.250 (9.221 ~ 13.279)	**<0.001**

Abbreviations: BMI, body mass index; MD, mean difference; CI, confidence interval; SD, standard deviation; SBP, systolic blood pressure

## Discussion

This retrospective study indicated that BMI < 18.5 kg/m²was identified as an independent risk factor for increased RBC transfusion rate during perioperative periods in patients who underwent isolated CABG surgery with or without CPB. Moreover, among patients who underwent on-pump CABG, BMI < 18.5 kg/m² was specifically associated with an increased rate of plasma and platelet transfusions. Underweight patients demonstrated a higher risk of re-intubation, re-operation, and 30-day all-cause mortality, which was observed solely in the on-pump group of the original cohort. Post-hoc analysis indicated that these discrepancies in clinical outcomes might be attributed to underlying malnutrition and a more significant decrease in blood pressure after induction in patients with a BMI < 18.5 kg/m².

CABG surgery is a commonly performed cardiovascular surgical intervention, with its prevalence escalating in response to the aging demographic in China [20]. Consequently, the identification of risk factors has been acknowledged as a crucial way to enhancing patient counseling, surgical planning, and targeted initiatives for improving the quality of care.

Certain easily obtainable preoperative parameters offer the potential to predict perioperative transfusion and clinical outcomes. Among these, BMI is acknowledged by the WHO as a standardized and valuable demographic indicator of weight status in large-scale adult nutritional status surveys. Moreover, it has been evaluated as a potential risk factor for adverse events following various surgical procedures [21]. Nevertheless, the relationship between BMI and perioperative blood transfusion and adverse events during cardiovascular surgery remains uncertain [22, 23]. Studies have suggested that obese patients undergoing cardiac surgery exhibit relatively lower rates of blood transfusion and adverse events, possibly attributed to increased estrogen levels in adipose tissue, which have a coagulant effect on the clotting system [24]. A study hypothesized that the obese state is characterized by chronic inflammation due to the secretion of adipokines and cytokines by adipose tissue and adipose tissue-derived macrophages [25]. This inflammatory state, triggered by nutrient excess, engages signaling pathways similar to those involved in the adaptive response to injury. Overweight and mildly obese patients are better equipped to mount an appropriate inflammatory response during surgery compared to underweight and morbidly obese patients, who experience hyperbolic inflammatory responses and metabolic dysfunction, leading to poorer outcomes. Despite several studies suggesting obesity as a predictive factor for perioperative adverse events or mortality, the focus has been primarily on the upper end of the BMI range [26, 27]. This emphasis is influenced by the higher prevalence of cardiovascular disease and increasing obesity rates in developed countries, leading to a lack of data on underweight individuals undergoing such procedures.

Recent studies have investigated the impact of low body weight on perioperative outcomes in cardiovascular surgery [28, 29]. However, these studies face limitations such as the rarity of underweight patients, resulting in small sample sizes for analysis. Additionally, studies often combine underweight patients with those having a normal BMI, which hampers the examination of underweight as a distinct BMI group. Moreover, the focus on on-pump CABG and the scarcity of studies on off-pump CABG may obscure the true influence of low body weight on outcomes. Therefore, a more nuanced classification of the population is necessary to comprehensively understand the effect of low BMI on surgical outcomes. In a recent study by Zheng et al., a novel predictive model was developed to assess the risk of in-hospital mortality following CABG in China [30]. Unlike existing risk prediction models such as the STS risk prediction model and EuroSCORE II, this new model incorporated a previously unconsidered factor: BMI < 18.5 kg/m². Notably, the newly proposed model demonstrated superior accuracy compared to EuroSCORE II and SinoSCORE, highlighting the significance of low body weight as a noteworthy preoperative indicator in the Chinese population, garnering increasing attention.

A BMI < 18.5 kg/m² has been identified as an independent risk factor for increased perioperative RBC transfusion in patients undergoing on-pump CABG, which is consistent with existing literature [31]. This association can be attributed to the increased hemodilution effect of CPB in underweight patients. Notably, our study also revealed a novel positive correlation between underweight and RBC transfusion rates in patients undergoing off-pump CABG (OR = 3.630, 95% CI = 1.326–9.937, p = 0.012), with significantly higher transfusion volumes compared to the normal BMI group (1.26 ± 1.97 vs. 0.44 ± 1.08, p = 0.003). Post-hoc analysis indicated that underweight patients experienced a significant reduction in SBP after anesthesia induction, potentially due to reduced vascular elasticity. Consistent with previous studies on the relationship between BMI and arteriosclerosis in the Chinese population, our study found a negative correlation between BMI and arteriosclerosis after accounting for confounding factors, indicating that higher BMI levels are associated with improved arterial elasticity after adjusting for confounding variables [32, 33]. Arterial elasticity emerged as an independent risk factor for hypotension following anesthesia induction, and a substantial decrease in blood pressure during induction typically necessitated a larger volume of fluids to maintain blood pressure and ensure sufficient tissue and organ perfusion [34–37]. As a result, underweight patient may have received as a result fluid volumes during surgery, potentially leading to hemodilution.

Regarding plasma and platelet utilization, different outcomes were observed between the on-pump and off-pump groups. Underweight patients exhibited significantly increased rates of plasma and platelet transfusion in the on-pump group, while no difference was observed in the off-pump group. This discrepancy can be attributed to variances in surgical techniques, aligning with prior research that suggests it may be linked to hemodilution, hemolysis, and suppression of platelet function induced by systemic heparinization during CPB in the on-pump group [38, 39]. This accounts for the absence of observed differences in plasma and platelet transfusion rates within the off-pump group.

In the analysis of clinical outcomes in this study, there was no evidence of an increased risk of perioperative adverse events associated with a BMI < 18.5 kg/m² in the matched cohort (p > 0.05). However, in the on-pump group of original cohort, BMI < 18.5 kg/m² was identified as an independent risk factor for re-intubation, re-operation and 30-day all-cause mortality (OR = 5.365, 95% CI = 1.159 ~ 24.833, p = 0.032; OR = 4.650, 95% CI = 1.019 ~ 21.210, p = 0.047; OR = 10.325, 95% CI = 2.011 ~ 53.020, p = 0.005). This could potentially be attributed to the significant differences in prealbumin, albumin, and A/G ratio that were identified between the two groups during the post-hoc analysis. Surgical trauma induces stress responses that can result in hyperglycemia and protein catabolism [40, 41]. Patients undergoing cardiac surgery, especially those requiring CPB, experience significant surgical trauma and are prone to have life-threatening complications associated with systemic inflammatory response syndrome [42, 43]. Nutritional biomarkers such as albumin, globulin, and the A/G ratio are indicators of inflammation and may also reflect muscle mass [44]. Evaluating malnutrition using BMI and these protein markers is important, as malnutrition is associated with increased postoperative complications, decreased respiratory muscle function, higher infection rates, prolonged hospital stays, and increased 30-day mortality [45–47]. Therefore, providing early nutritional support, particularly for patients with low BMI, is recommended for cardiac surgery patients.

Our study has several limitations. Firstly, the rarity of underweight individuals resulted in a significant difference in case numbers between the two groups, leading to a loss of observational values following propensity score matching. Additionally, as this study was conducted in a single center, the generalizability of the findings may be affected, necessitating further multicenter prospective studies to validate our results and provide stronger evidence. Secondly, the quality of the results relies on the accuracy of the data, with clinical diagnoses of comorbidities extracted from the medical record system. The lack of information on disease severity may contribute to residual or confounding results. Moreover, there may be unmeasured confounders not accounted for, potentially influencing the association between BMI and outcomes. Furthermore, the analysis did not include blood loss due to missing data exceeding 40%. Lastly, the absence of specific time information for transfusions prevented differentiation between preoperative, intraoperative, and postoperative periods.

## Conclusion

In conclusion, our study demonstrated that BMI < 18.5 kg/m² was identified as an independent risk factor for increased perioperative RBC transfusion rate in patients who underwent isolated CABG with or without CPB. Only on-pump underweight patients in the original cohort exhibited an increased risk for re-intubation, re-operation, and 30-day all-cause mortality. These findings underscore the importance of recognizing the unique challenges faced by underweight individuals in the perioperative setting and highlight the need for tailored interventions and close monitoring to optimize outcomes. Further research is warranted to validate our findings and explore strategies to improve outcomes for this specific patient population.

## Data Availability

The datasets used and/or analyzed during the current study are available from the corresponding author on reasonable request.

## Abbreviations

BMI: body mass index
CABG: coronary artery bypass grafting
RBC: red blood cell
CVD: cardiovascular disease
CPB: cardiopulmonary bypass
ICD-9: International Classification of Diseases, 9th Revision
AKI: acute kidney injury
WHO: World Health Organization
WGOC: Work Group on Obesity in China
SBP: systolic blood pressure
IQR: interquartile range
COPD: chronic obstructive pulmonary disease
CHF: congestive heart failure
A/G: albumin/globulin
